# Clinical Epidemiology of Bocavirus, Rhinovirus, Two Polyomaviruses and Four Coronaviruses in HIV-Infected and HIV-Uninfected South African Children

**DOI:** 10.1371/journal.pone.0086448

**Published:** 2014-02-03

**Authors:** Marta C. Nunes, Zachary Kuschner, Zelda Rabede, Richard Madimabe, Nadia Van Niekerk, Jackie Moloi, Locadiah Kuwanda, John W. Rossen, Keith P. Klugman, Peter V. Adrian, Shabir A. Madhi

**Affiliations:** 1 Department of Science and Technology/National Research Foundation: Vaccine Preventable Diseases, Johannesburg, South Africa; 2 Medical Research Council: Respiratory and Meningeal Pathogens Research Unit, Faculty of Health Sciences, University of the Witwatersrand, Johannesburg, South Africa; 3 Stony Brook School of Medicine, Stony Brook University, Stony Brook, New York, United States of America; 4 Molecular and Immunology Division, BioMérieux, Johannesburg, South Africa; 5 Department of Medical Microbiology, University Medical Center Groningen, University of Groningen, Groningen, The Netherlands; 6 Hubert Department of Global Health, Rollins School of Public Health and Division of Infectious Diseases, Emory University School of Medicine, Atlanta, Georgia, United States of America; 7 National Institute for Communicable Diseases: a division of National Health Laboratory Service, Sandringham, South Africa; Columbia University, United States of America

## Abstract

**Background:**

Advances in molecular diagnostics have implicated newly-discovered respiratory viruses in the pathogenesis of pneumonia. We aimed to determine the prevalence and clinical characteristics of human bocavirus (hBoV), human rhinovirus (hRV), polyomavirus-WU (WUPyV) and –KI (KIPyV) and human coronaviruses (CoV)-OC43, -NL63, -HKU1 and -229E among children hospitalized with lower respiratory tract infections (LRTI).

**Methods:**

Multiplex real-time reverse-transcriptase polymerase chain reaction was undertaken on archived nasopharyngeal aspirates from HIV-infected and –uninfected children (<2 years age) hospitalized for LRTI, who had been previously investigated for respiratory syncytial virus, human metapneumovirus, parainfluenza I–III, adenovirus and influenza A/B.

**Results:**

At least one of these viruses were identified in 274 (53.0%) of 517 and in 509 (54.0%) of 943 LRTI-episodes in HIV-infected and -uninfected children, respectively. Human rhinovirus was the most prevalent in HIV-infected (31.7%) and –uninfected children (32.0%), followed by CoV-OC43 (12.2%) and hBoV (9.5%) in HIV-infected; and by hBoV (13.3%) and WUPyV (11.9%) in HIV-uninfected children. Polyomavirus-KI (8.9% vs. 4.8%; p = 0.002) and CoV-OC43 (12.2% vs. 3.6%; p<0.001) were more prevalent in HIV-infected than –uninfected children. Combined with previously-tested viruses, respiratory viruses were identified in 60.9% of HIV-infected and 78.3% of HIV-uninfected children. The newly tested viruses were detected at high frequency in association with other respiratory viruses, including previously-investigated viruses (22.8% in HIV-infected and 28.5% in HIV–uninfected children).

**Conclusions:**

We established that combined with previously-investigated viruses, at least one respiratory virus was identified in the majority of HIV-infected and HIV-uninfected children hospitalized for LRTI. The high frequency of viral co-infections illustrates the complexities in attributing causality to specific viruses in the aetiology of LRTI and may indicate a synergetic role of viral co-infections in the pathogenesis of childhood LRTI.

## Introduction

Pneumonia is a leading cause of mortality in children under 5 years age worldwide, including in HIV-infected children [1]–[3]. The aetiology of childhood pneumonia may include infection with bacteria and/or respiratory viruses. Although respiratory viruses are more frequently identified than bacteria in children with pneumonia, this may be biased by lack of availability of sensitive and specific tests for diagnosing bacterial causes of pneumonia [4]. Furthermore, respiratory viral infections may heighten the susceptibility to developing a super-imposed bacterial infection resulting in severe pneumonia [5], [6]. Traditionally, respiratory viruses that have been associated with lower respiratory tract infections (LRTI) include respiratory syncytial virus (RSV), parainfluenza viruses I–III (PIV I–III), influenza viruses A/B and adenovirus. Two human coronaviruses (CoV), OC43 (CoV-OC43) and 229E (CoV-229E) were initially identified as causes of upper respiratory tract infections (URTI) in the 1960s using classical culture methods [7], [8]. More recently, advances in molecular diagnostics have resulted in the discovery of other respiratory viruses which have also been associated with LRTI. Included among these are human metapneumovirus (hMPV) [9], human bocavirus (hBoV) [10], human coronavirus NL63 (CoV-NL63) [11] and HKU1 (CoV-HKU1) [12] and WU and KI polyomaviruses (WUPyV, KIPyV) [13]–[15]. Also, human rhinovirus (hRV), which was previously mainly associated with mild URTI, has increasingly been implicated in having a role in the pathogenesis of LRTI and asthma exacerbations [16], [17].

Due to impaired humoral and cell-mediated immunity, HIV infection in children has been described as a risk factor for severe illness and mortality caused by respiratory-viral associated LRTI, such as RSV, hMPV and influenza virus [18], [19]. There are, however, limited data on the role of other respiratory viruses, including the more recently-discovered viruses which occur as single or co-infecting pathogens in HIV-infected children hospitalized with LRTI, and of these studies, most have small sample sizes [20]–[22].

The aim of this study was to identify the prevalence of hBoV, hRV, WUPyV, KIPyV, CoV-OC43, CoV-NL63, CoV-HKU1 and CoV-229E among HIV-infected and -uninfected children who were hospitalized for LRTI using real-time reverse transcriptase–polymerase chain reaction (RT-PCR). The study-cohort had been previously investigated for RSV, influenza A/B, PIV I-III and adenovirus by immunofluorescence assay and hMPV by nested-PCR as described [5], [23].

## Methods

### Ethics Statement

The main 9-valent pneumococcal conjugated vaccine (PCV9) efficacy trial and subsequent retrospective analysis of study participants were approved by the Human Research Ethics Committee (Medical) of the University of the Witwatersrand. The main study did not have a clinical trials number since it was undertaken prior to registration of clinical trials having been made mandatory. Signed informed consent was obtained from the parent/legal guardians of all the study participants as part of the main trial. The Ethics Committee did not require additional consent for this study.

### Study population

This was a retrospective study of children who participated in a phase III trial in South Africa which investigated the efficacy of a PCV9 as described [5], [24]. Briefly, 39836 children recruited from March 1998 to October 2000 were randomized (1∶1) to receive 3 doses of either PCV9 or placebo. Vaccination occurred at a mean of 6.6±1.2 (± standard deviation), 11.2±2.5, and 15.9±3.9 weeks of age [24]. Hospital-based surveillance for all-cause hospitalization was undertaken at Chris Hani-Baragwanath Hospital, the only public hospital in the study community. Hospitalized children had their signs and symptoms recorded and underwent HIV testing according to the study protocol [24]. Nasopharyngeal aspirates (NPA) were obtained for respiratory viral studies from children hospitalized with LRTI [5], and archived from January 2000 onward. In the present study only NPA collected from 1^st^ February 2000 to 31^st^ January 2002 from children less than 2 years old were analysed. If a child had recurrent LRTI hospitalizations, only NPA collected at least 28 days apart were included in the analysis. Blood cultures were performed with the use of an automated blood-culture system (BacT-Alert, Organon Teknika).

### Viral testing

Total nucleic acids were extracted from archived NPA using a NucliSENS easyMAG platform (bioMerieux), and eluted in a final volume of 60 µl of elution buffer [25]. RNA was reverse transcribed with High Capacity cDNA Reverse Transcriptase (Invitrogen, Life Technologies) and primed with oligo-dT primers (Invitrogen, Life Technologies). Real-time PCR was done in an ABI 7500 RT-PCR system (Applied Biosystems, Life Technologies), reactions were performed in 20 µl using TaqMan Universal PCR Master Mix (Applied Biosystems, Life Technologies) and the primers and probes listed in Table S1.

Five duplex RT-PCR reactions, targeting the 8 respiratory viruses, were developed. Internal controls (the human genes: ribonucleoprotein and glyceraldehyde-3-phosphate dehydrogenase; or the spiked viruses: lambda and Newcastle Disease Virus) were included to check the efficiency of the extraction step and to detect the presence of PCR inhibitors. Positive controls were included in each experiment.

### Study-specific definitions

The clinical definitions used in this study are the same as previously described [5]. Briefly, LRTI was defined as any episode with clinical diagnosis of pneumonia or bronchiolitis done by a study physician. Children with LRTI were categorized as having clinical pneumonia if they had evidence of alveolar consolidation on chest x-ray (CXR-AC) or if they fulfilled the clinical diagnosis of LRTI without wheeze on chest auscultation but had rales and/or bronchial breathing. Children were categorized as having bronchiolitis in the presence of wheezing on chest auscultation and in the absence of documented CXR-AC or bronchial breathing on chest wall auscultation. A clinical diagnosis of WHO severe/very severe pneumonia was made if the child had a cough <14 days in duration and lower chest wall in-drawing and/or any of the following signs and symptoms of severe pneumonia: feeding difficulties, convulsions, central cyanosis, or encephalopathy. The previously developed respiratory index of severity in children (RISC) score was used to compare disease severity between single and multiple viral infections [26].

### Statistical analysis

Demographic, clinical and laboratory characteristics at admission were compared between HIV-infected and –uninfected children, chi-square or Fisher's exact tests were used to compare the distribution of categorical variables and mean or median of continuous variables were compared by two-tailed Student t-test or Mann-Whitney test, respectively. Viral prevalence was compared between HIV groups and clinical and laboratory characteristics were compared between episodes with single and multiple viral detection using multivariate logistic regression models adjusted for study intervention arm (i.e. whether received PCV9 or placebo), year of sampling, detection of a virus previously-tested and age. The results are presented as adjusted odds ratios (aOR) with 95% confidence intervals (95% CI) and p-values. A p-value<0.05 was considered significant. Analyses were performed using STATA version 11.0 (Statacorp, Texas, USA).

## Results

### Study population

During the follow-up period included in this analysis, there were a total of 2147 hospitalizations for LRTI, including 2094 (97.5%) in which NPA had been collected. Of the initial collected samples 69.7% were available for RT–PCR analysis. The same proportion of samples were available for RT–PCR from PCV9-recipients (69.2%) and placebo-recipients (70.2%; p = 0.621), from HIV-infected (78.2% vs. 73.1%; p = 0.130) and HIV-uninfected children (65.5% vs. 68.6%; p = 0.218); Table 1. A lower proportion of samples was, however, available for RT-PCR analysis (65.9% [1000/1518]) from the first period (February 2000–January 2001) compared to the second period (February 2001–January 2002) (79.9% [460/576]; p<0.001) of the study.

**Table 1:** Number of specimens analysed in the current study and total specimens collected.

	Overall				HIV-infected				HIV-uninfected			
	Overall	PCV9	Placebo	p-value^1^	Overall	PCV9	Placebo	p-value^1^	Overall	PCV9	Placebo	p-value^1^
Visits according to study criteria^2^	2147	1038	1109		712	310	402		1435	728	707	
Nasopharyngeal aspirate done	2094 (97.5)	1010 (97.3)	1084 (97.7)	0.508	687 (96.5)	297 (95.8)	390 (97.0)	0.385	1407 (98.1)	713 (97.9)	694 (98.2)	0.762
Viral RT-PCR performed in this study	1460 (69.7)	699 (69.2)	761 (70.2)	0.621	517 (75.3)	232 (78.2)	285 (73.1)	0.130	943 (67.0)	467 (65.5)	476 (68.6)	0.218

1 : Chi-square test comparing 9-valent pneumococcal conjugated vaccine (PCV9) and placebo arms.

2 : From the full database: patients <2 years old, with diagnosis of lower respiratory tract infections, hospitalized between 1 February 2000 and 31 January 2002, only 1 visit per patient within 28 days.

Children from whom NPA were available for RT–PCR testing compared to those in whom samples were unavailable were older (median age: 10 vs. 8 months; p<0.001), were 1.3-fold less likely to have tested positive for one of the previously-tested respiratory viruses (33.3% vs. 42.3%; p<0.001), had a higher prevalence of cyanosis (11.4% vs. 8.1%; p = 0.025), higher evidence of CXR-AC (26.6% vs. 22.1%; p = 0.041), higher C-reactive protein (CRP) levels (median: 15 vs. 12 mg/l; p = 0.003) and higher procalcitonin (PCT) concentration (median: 0.26 vs. 0.15 ng/ml; p = 0.006); Table S2. There were no other demographic, clinical or laboratory differences observed between the LRTI-episodes with NPA available versus unavailable for RT-PCR.

A total of 517 NPA samples from HIV-infected children were analysed by RT–PCR, including 45.0% from PCV9-recipients and 55.0% from placebo-recipients. Among HIV-uninfected children, 943 specimens were available for RT-PCR analysis, including 49.5% from PCV9-recipients and 50.5% from placebo-recipients. On admission HIV-infected children compared to HIV-uninfected were younger (median age: 9 vs. 11 months; p<0.001), more frequently presented with cyanosis (23.8% vs. 4.6%; p<0.001), had lower mean oxygen saturation (89.8% vs. 92.2%; p<0.001), had a higher median respiratory rate (54 vs. 48 breaths per minute; p<0.001), were more likely to present as pneumonia (88.8% vs. 49.6%; p<0.001) than bronchiolitis, had higher CRP (18 vs. 14 mg/l; p = 0.007) and PCT levels (0.47 vs. 0.17 ng/ml; p<0.001), had a longer hospital stay (4 vs. 1 median days; p<0.001), had a higher case-fatality rate (17.8% vs. 0.95%; p<0.001) and more frequently had bacteraemia (7.8% vs. 2.7; p<0.001); Table 2.

**Table 2:** Demographic, clinical and laboratory features of children hospitalized for lower respiratory tract infection and in whom samples were available for the current study.

		OverallN = 1460PVC9 = 699Placebo = 761	HIV-infectedN = 517PCV9 = 232Placebo = 285	HIV-uninfectedN = 943PCV9 = 467Placebo = 476	p-value^1^
**Demographic characteristics**					
	Overall	10 (1–24)	9 (1–24)	11 (1–23)	<0.001
Median age in months,	PCV9	10 (1–23)	8 (1–23)	11 (1–23)	0.018
(range)	Placebo	10 (1–24)	9 (1–24)	11(1–23)	0.009
	p-value^2^	0.612	0.706	0.512	
	Overall	836 (57.3)	284 (54.9)	552 (58.5)	0.194
Male^3^, N (%)	PCV9	423 (60.5)	137 (59.1)	286 (61.2)	0.577
	Placebo	413 (54.3)	147 (51.6)	266 (55.9)	0.249
	p-value^2^	0.015	0.089	0.095	
**Clinical characteristics**					
	Overall	37.3 (0.96)	37.3 (0.93)	37.3 (1.0)	0.895
Mean axillary	PCV9	37.2 (0.80)	37.2 (0.81)	37.3 (0.79)	0.675
temperature^4^, °C (SD)	Placebo	37.4 (1.1)	37.5 (1.0)	37.4 (1.1)	0.768
	p-value^2^	0.005	0.032	0.05	
	Overall	222 (27.3)	85 (29.7)	137 (26.0)	0.255
Axillary temperature ≥38°C,	PCV9	89 (23.3)	29 (23.0)	60 (23.4)	0.927
N (%)	Placebo	133 (30.9)	56 (35.0)	77 (28.4)	0.153
	p-value^2^	0.016	0.028	0.193	
	Overall	1054 (72.6)	369 (71.9)	685 (73.0)	0.654
Fever^5^, N (%)	PCV9	488 (70.2)	155 (67.4)	333 (71.6)	0.252
	Placebo	566 (74.9)	214 (75.6)	352 (74.4)	0.713
	p-value^2^	0.047	0.039	0.333	
	Overall	624 (42.9)	209 (40.7)	415 (44.1)	0.205
Vomit^6^, N (%)	PCV9	275 (39.5)	85 (37.0)	190 (40.8)	0.333
	Placebo	349 (46.0)	124 (43.7)	225 (47.4)	0.321
	p-value^2^	0.014	0.124	0.042	
	Overall	45 (3.1)	9 (1.8)	36 (3.9)	0.029
Seizures^7^, N (%)	PCV9	23 (3.3)	4 (1.7)	19 (4.1)	0.102
	Placebo	22 (2.9)	5 (1.8)	17 (3.6)	0.152
	p-value^2^	0.661	0.973	0.685	
	Overall	165 (11.4)	122 (23.8)	43 (4.6)	<0.001
Cyanosis^8^, N (%)	PCV9	82 (11.8)	59 (25.5)	23 (5.0)	<0.001
	Placebo	83 (11.0)	63 (22.3)	20 (4.2)	<0.001
	p-value^2^	0.617	0.397	0.584	
	Overall	91.3 (5.7)	89.8 (5.5)	92.2 (5.5)	<0.001
Mean oxygen saturation %	PCV9	91.1 (6.6)	89.0 (5.8)	92.1 (6.8)	<0.001
on room air, (SD)	Placebo	91.6 (4.6)	90.3 (5.2)	92.4 (4.0)	<0.001
	p-value^2^	0.072	0.008	0.395	
	Overall	358 (24.8)	223 (44.1)	135 (14.4)	<0.001
Mean oxygen saturation	PCV9	175 (25.4)	107 (47.8)	68 (14.7)	<0.001
on room air <90%^9^, N (%)	Placebo	183 (24.3)	116 (41.1)	67 (14.2)	<0.001
	p-value^2^	0.619	0.135	0.852	
	Overall	50 (22–130)	54 (24–130)	48 (22–96)	<0.001
Median respiratory rate	PCV9	50 (24–130)	58 (24–130)	48 (24–90)	<0.001
breaths per minute^10^, (range)	Placebo	48 (22–100)	52 (24–100)	48 (22–96)	<0.001
	p-value^2^	0.167	0.040	0.439	
	Overall	927 (63.5)	459 (88.8)	468 (49.6)	<0.001
Clinical pneumonia, N (%)	PCV9	419 (59.9)	207 (89.2)	212 (45.4)	<0.001
	Placebo	508 (66.8)	252 (88.4)	256 (53.8)	<0.001
	p-value^2^	0.007	0.774	0.010	
	Overall	763 (84.4)	300 (89.3)	463 (81.5)	0.002
WHO severe pneumonia^11^,	PCV9	352 (81.5)	143 (91.1)	209 (76.0)	<0.001
N (%)	Placebo	411 (87.1)	157 (87.7)	254 (86.7)	0.749
	p-value^2^	0.021	0.319	0.001	
	Overall	628 (43.1)	90 (17.4)	538 (57.2)	<0.001
Wheezing^12^, N (%)	PCV9	317 (45.4)	34 (14.7)	283 (60.7)	<0.001
	Placebo	311 (40.9)	56 (19.7)	255 (53.7)	<0.001
	p-value^2^	0.083	0.136	0.029	
	Overall	128 (8.8)	95 (18.4)	33 (3.5)	<0.001
Bronchial breathing, N (%)	PCV9	57 (8.2)	41 (17.7)	16 (3.4)	<0.001
	Placebo	71 (9.3)	54 (19.0)	17 (3.6)	<0.001
	p-value^2^	0.428	0.710	0.903	
	Overall	19 (1.3)	3 (0. 59)	16 (1.7)	0.072
Mechanical ventilation^13^,	PCV9	7 (1.0)	1 (0.43)	6 (1.3)	0.283
N (%)	Placebo	12 (1.6)	2 (0.72)	10 (2.1)	0.137
	p-value^2^	0.329	0.679	0.334	
	Overall	342 (26.6)	189 (43.2)	153 (18.1)	<0.001
CXR-AC^14^, N (%)	PCV9	152 (25.0)	84 (42.0)	68 (16.7)	<0.001
	Placebo	190 (28.1)	105 (44.1)	85 (19.4)	<0.001
	p-value^2^	0.215	0.656	0.308	
	Overall	2 (0–49)	4 (0–49)	1 (0–42)	<0.001
Median hospital stay	PCV9	2 (0–49)	5 (0–49)	1 (0–24)	<0.001
in days, (range)	Placebo	2 (0–49)	3 (0–49)	1 (0–42)	<0.001
	p-value^2^	0.949	0.070	0.378	
	Overall	101 (6.9)	92 (17.8)	9 (0.95)	<0.001
Deaths due to LRTI, N (%)	PCV9	50 (7.2)	46 (19.8)	4 (0.86)	<0.001
	Placebo	51 (6.7)	46 (16.1)	5 (1.1)	<0.001
	p-value^2^	0.734	0.276	0.759	
**Laboratory and other investigations**					
	Overall	15 (1–464)	18 (1–464)	14 (1–446.2)	0.007
Median CRP mg/l^15^,	PCV9	15 (1–446.2)	18 (1–443.1)	13 (1–446.2)	0.077
(range)	Placebo	16.9 (1–464)	19 (1–464)	15 (1–363.2)	0.036
	p-value^2^	0.102	0.258	0.244	
	Overall	313 (30.2)	146 (35.5)	167 (26.8)	0.003
CRP ≥40 mg/l, N (%)	PCV9	137 (27.4)	61 (31.3)	76 (24.9)	0.120
	Placebo	176 (32.9)	85 (39.4)	91 (28.5)	0.009
	p-value^2^	0.054	0.088	0.309	
	Overall	0.26 (0.01–302)	0.47 (0.01–302)	0.17 (0.01–150.1)	<0.001
Median PCT ng/ml^16^,	PCV9	0.23 (0.01–175)	0.4 (0.01–175)	0.14 (0.01–119.9)	<0.001
(range)	Placebo	0.29 (0.01–302)	0.57 (0.01–302)	0.19 (0.08–150.1)	<0.001
	p-value^2^	0.138	0.233	0.309	
	Overall	177 (20.5)	87 (28.1)	90 (16.2)	<0.001
PCT ≥2 ng/ml, N (%)	PCV9	78 (18.6)	38 (25.5)	40 (14.8)	0.007
	Placebo	99 (22.3)	49 (30.4)	50 (17.6)	0.005
	p-value^2^	0.205	0.334	0.363	
	Overall	13 (1–77.6)	12.5 (2.8–77.6)	13.6 (1–64.7)	0.307
Median WCC ×10^9^	PCV9	12 (1–37.4)	11 (2.8–29.5)	12.2 (1–37.4)	0.502
cells/ml^17^, (range)	Placebo	13.6 (3.2–77.6)	13 (3.2–77.6)	14.5 (4.3–64.7)	0.371
	p-value^2^	0.037	0.197	0.083	
	Overall	62 (4.5)	38 (7.8)	24 (2.7)	<0.001
Bacteraemia^18^, N (%)	PCV9	28 (4.3)	12 (5.5)	16 (3.7)	0.288
	Placebo	34 (4.8)	26 (9.8)	8 (1.8)	<0.001
	p-value^2^	0.657	0.077	0.086	

In parenthesis percent of number studied, unless otherwise indicated.

1 : Chi-square or Fischer test and two-tailed Student t-test or Mann-Whitney test comparing HIV groups.

2 : Chi-square or Fischer test and two-tailed Student t-test or Mann-Whitney test comparing 9-valent pneumococcal conjugated vaccine (PCV9) and placebo arms.

3 Gender: HIV-infected overall female vs. male: 45.1% vs. 54.9%, p = 0.001; HIV-infected PCV9 female vs. male: 41.0% vs. 59.1%, p<0.001; HIV-infected placebo female vs. male: 48.4% vs. 51.6%, p = 0.451; HIV-uninfected overall female vs. male: 41.5% vs. 58.5%, p<0.001; HIV-uninfected PCV9 female vs. male: 38.8% vs. 61.2%, p<0.001; HIV-uninfected placebo female vs. male: 44.1% vs. 55.9%, p<0.001.

4 Axillary temperature: 813 patients with available information; HIV-infected 286; HIV-uninfected 527.

5 Fever: 1451 patients with available information; HIV-infected 513; HIV-uninfected 938.

6 Vomit: 1455 patients with available information; HIV-infected 514; HIV-uninfected 941.

7 Seizures: 1447 patients with available information; HIV-infected 511; HIV-uninfected 936.

8 Cyanosis: 1454 patients with available information; HIV-infected 513; HIV-uninfected 941.

9 Oxygen saturation: 1441 patients with available information; HIV-infected 506; HIV-uninfected 935.

10 Respiratory rate: 1446 patients with available information; HIV-infected 515; HIV-uninfected 930.

11 World Health Organization (WHO) severe pneumonia definition: 904 patients with available information; HIV-infected 336; HIV-uninfected 568.

12 Wheezing: 1458 patients with available information; HIV-infected 517; HIV-uninfected 941.

13 Mechanical ventilation: 1439 patients with available information; HIV-infected 509; HIV-uninfected 930. At the time of the study it was current practice not to offer ventilation to severe AIDS patients.

14 Alveolar consolidation on chest x-ray (AC-CXR): 1285 patients with available information; HIV-infected 438; HIV-uninfected 847.

15 C-reactive protein (CRP): 1035 patients with available information; HIV-infected 411; HIV-uninfected 624.

16 Procalcitonin (PCT): 865 patients with available information; HIV-infected 310; HIV-uninfected 555.

17 White cell count (WCC): 209 patients with available information; HIV-infected 95; HIV-uninfected 114.

18 Bacteria infections: overall 1367 patients had blood specimens available for culture, 486 HIV-infected and 881 HIV-uninfected. Bacteria isolated from HIV-infected children included: *Streptococcus pneumoniae* (n = 16), *Escherichia coli* (n = 9), *Salmonella* sp (n = 5), *Streptococcus viridans* (n = 2), *Haemophilus influenzae* type b (n = 1), *Pseudomonas aeruginosa* (n = 1), *Haemophilus parainfluenzae* (n = 1), *Citrobacter freundii* (n = 1), *Staphylococcus aureus* (n = 1) and other *Streptococcus* (n = 1). Bacteria isolated from HIV-uninfected children included: *Streptococcus pneumoniae* (n = 8), *Haemophilus influenzae* type b (n = 3), *Streptococcus* viridans (n = 3), *Escherichia coli* (n = 2), *Klebsiella* sp. (n = 2), *Salmonella* sp (n = 1), *Streptococcus agalactiae* (n = 1), *Staphylococcus aureus* (n = 1), *Enterococcus faecalis* (n = 1), *Micrococcus* (n = 1) and *Neisseria meningitidis* (n = 1). Further 64 (62.7% of all bacteria isolated) and 107 (81.7% of all bacteria isolated) contaminants were cultured in HIV-infected and -uninfected children, respectively.

Of the 1460 specimens analysed, all but 13 (1 from HIV-infected and 12 from HIV-uninfected children) were previously tested for hMPV by nested-PCR [23] and 1458 were tested by an immunofluorescence assay for RSV, which if found negative, were further tested for influenza A/B, PIV I–III and adenovirus as described [5].

### Prevalence of newly-tested respiratory viruses

Among HIV-infected children, the RT-PCR viral panel was positive in 274 (53.0%) LRTI-episodes for at least one of the newly-tested viruses; Table 3. The prevalence of any of the newly-tested respiratory viruses was similar between PCV9- and placebo-recipients in HIV-infected children, except for WUPyV (11.2% vs. 6.3%; p = 0.047, respectively) and hBoV (12.5% vs. 7.0%; p = 0.034, respectively); Table S3. In HIV-infected children hRV was the most frequently detected virus (31.7%) followed by CoV-OC43 (12.2%), hBoV (9.5%), KIPyV (8.9%), WUPyV (8.5), CoV-NL63 (1.7%) and CoV-HKU1 (1.4%); Table 3. The newly-tested viruses were frequently identified as co-infecting viruses among HIV-infected children, including 49.4% of LRTI-episodes associated with hRV; Table 4. The most common viral co-infections with hRV included KIPyV (14.6%), WUPyV (11.6%), CoV-OC43 and hBoV (11.0%, each); Table 4. Among the 486 children on whom blood culture was done, bacteria were isolated on 38 (7.8%) occasions, 20 (52.6%) of which were associated with concomitant detection of one of the newly-tested viruses and 22 (57.9%) of any of the viruses.

**Table 3:** Viral prevalence of newly- or previously-tested viruses according to HIV status.

	Overall n (%)N = 1460	HIV-infected n (%)N = 517	HIV-uninfected n (%)N = 943	OR (95% CI)p-value	aOR^1^ (95% CI)p-value
hBoV	174 (11.9)	49 (9.5)	125 (13.3)	0.69 (0.48–0.97)0.034	0.69 (0.48–0.99)0.043
WUPyV	156 (10.7)	44 (8.5)	112 (11.9)	0.69 (0.48–1.00)0.047	0.66 (0.45–0.97)0.035
KIPyV	91 (6.2)	46 (8.9)	45 (4.8)	1.95 (1.27–2.98)0.002	2.14 (1.36–3.37)0.001
CoV-NL63	33 (2.3)	9 (1.7)	24 (2.6)	0.68 (0.31–1.47)0.326	0.65 (0.29–1.45)0.294
CoV-HKU1	22 (1.5)	7 (1.4)	15 (1.6)	0.85 (0.34–2.10)0.723	0.67 (0.26–1.69)0.391
CoV-OC43	97 (6.6)	63 (12.2)	34 (3.6)	3.71 (2.41–5.71)<0.001	3.67 (2.30–5.85)<0.001
CoV-229E	4 (0.27)	0	4 (0.42)	0.304^2^	-
hRV	466 (31.9)	164 (31.7)	302 (32.0)	0.99 (0.78–1.24)0.905	0.82 (0.64–1.05)0.111
RSV^3^	237 (16.3)	32 (6.2)	205 (21.8)	0.24 (0.16–0.35)<0.001	0.22^7^ (0.15–0.33)<0.001
Influenza A^3^	61 (4.2)	13 (2.5)	48 (5.1)	0.48 (0.26–0.90)0.021	0.50^7^ (0.27–0.94)0.031
PIV I–III^3^	58 (4.0)	14 (2.7)	44 (4.7)	0.57 (0.31–1.05)0.071	0.55^7^ (0.30–1.02)0.056
Adenovirus^3^	29 (2.0)	4 (0.77)	25 (2.7)	0.29 (0.10–0.83)0.021	0.27^7^ (0.09–0.79)0.016
hMPV^4^	107 (7.4)	17 (3.3)	90 (9.7)	0.32 (0.19–0.54)<0.001	0.31^7^ (0.18–0.53)<0.001
At least 1 newly-tested virus detected	783 (53.6)	274 (53.0)	509 (54.0)	0.96 (0.78–1.20)0.720	0.83 (0.66–1.05)0.117
At least 1 of any tested viruses detected^5^	1053 (72.1)	315 (60.9)	738 (78.3)	0.43 (0.34–0.55)<0.001	0.43^7^ (0.34–0.55)<0.001
Viral co-infections^5^^,^ 6	389 (26.6)	118 (22.8)	271 (28.7)	0.73 (0.57–0.94)0.015	0.74^7^ (0.57–0.95)0.018
Newly- tested virus single infection^8^	396 (27.1)	156 (30.2)	240 (25.5)	1.27 (1.00–1.61)0.052	1.30^7^ (1.02–1.65)0.033
Bacteraemia if positive for at least 1 of the newly- tested viruses	32/733 (4.4)	20/260 (7.7)	12/473 (2.5)	3.20 (1.54–6.66)0.002	3.49 (1.63–7.48)0.001

1 : Adjusted odds ratio (aOR) adjusted for age, year of sampling, detection of viruses previously-tested and whether received 9-valent pneumococcal conjugate vaccine or placebo.

2 : p-value not adjusted.

3 : Previously-tested by immunofluorescence assay.

4 : Previously-tested by nested PCR.

5 : Including viruses previously-tested by immunofluorescence assay (RSV, Influenza A, PIV I–III and adenovirus) and nested-PCR (hMPV).

6 : All multiple infections included at least one newly-tested virus except in 2 HIV-uninfected children. Viral co-infections with at least one newly-tested virus in HIV-infected children 118 (22.8%) and in HIV-uninfected children 269 (28.5%).

7 : Not adjusted for detection of viruses previously-tested.

8 : Single infections with a newly-tested virus no other virus, newly- or previously-tested, was detected.

OR: odds ratio.

hBoV: human bocavirus.

WUPyV: polyomavirus-WU.

KIPyV: polyomavirus-KI.

CoV-NL63: coronavirus NL63.

CoV-HKU1: coronavirus HKU1.

CoV-OC43: coronavirus OC43.

CoV-229E: coronavirus 229E.

hRV: human rhinovirus.

RSV: respiratory syncytial virus.

PIV: parainfluenza viruses.

hMPV: human metapneumovirus.

**Table 4:** Respiratory viruses co-infections in HIV-infected children hospitalized with lower respiratory tract infections.

	hBoV	WUPyV	KIPyV	CoV-NL63	CoV-HKU1	CoV-OC43	hRV	RSV^4^	hMPV^5^	PIV^4^	Influenza A^4^	Adenovirus^4^
Positive samples^1^	49 (9.5)	44 (8.5)	46 (8.9)	9 (1.7)	7 (1.4)	63 (12.2)	164 (31.7)	32 (6.2)	17 (3.3)	14 (2.7)	13 (2.5)	4 (0.77)
Single infections^2^	15 (30.6)	16 (36.4)	11 (23.9)	0	4 (57.1)	27 (42.9)	83 (50.6)	20 (62.5)	6 (35.3)	8 (57.1)	4 (30.8)	3 (75.0)
Co-infections with^3^:												
hBoV	-	3 (6.8)	4 (8.7)	0	0	8 (12.7)	18 (11.0)	3 (9.4)	3 (17.6)	1 (7.1)	1 (7.7)	0
WUPyV	3 (6.1)	-	7 (15.2)	1 (11.1)	2 (28.6)	4 (6.3)	19 (11.6)	3 (9.4)	2 (11.8)	0	1 (7.7)	0
KIPyV	4 (8.2)	7 (15.9)	-	2 (22.2)	1 (14.3)	5 (7.9)	24 (14.6)	5 (15.6)	3 (17.6)	1 (7.1)	0	0
CoV-NL63	0	1 (2.3)	2 (4.3)	-	0	1 (1.6)	6 (3.7)	0	1 (5.9)	0	0	0
CoV-HKU1	0	2 (4.5)	1 (2.2)	0	-	0	0	0	0	0	0	0
CoV-OC43	8 (16.3)	4 (9.1)	5 (10.9)	1 (11.1)	0	-	18 (11.0)	1 (3.1)	2 (11.8)	3 (21.4)	4 (30.8)	0
hRV	18 (36.7)	19 (43.2)	24 (52.2)	6 (66.7)	0	18 (28.6)	-	8 (25.0)	6 (35.3)	2 (14.3)	6 (46.2)	1 (25.0)
RSV^4^	3 (6.1)	3 (6.8)	5 (10.9)	0	0	1 (1.6)	8 (4.9)	-	0	0	0	0
hMPV^5^	3 (6.1)	2 (4.5)	3 (6.5)	1 (11.1)	0	2 (3.2)	6 (3.7)	0	-	0	0	0
PIV^4^	1 (2.0)	0	1 (2.2)	0	0	3 (4.8)	2 (1.2)	0	0	-	0	0
Influenza A^4^	1 (2.0)	1 (2.3)	0	0	0	4 (6.3)	6 (3.7)	0	0	0	-	0
Adenovirus^4^	0	0	0	0	0	0	1 (0.6)	0	0	0	0	0
1 viruses^6^	28 (57.1)	17 (38.6)	21 (45.7)	7 (77.8)	3 (42.9)	27 (42.9)	58 (35.4)	7 (21.9)	5 (29.4)	5 (35.7)	7 (53.8)	1 (25.0)
2 viruses^6^	5 (10.2)	8 (18.2)	11 (23.9)	2 (22.2)	0	8 (12.7)	19 (11.6)	2 (6.3)	6 (35.3)	1 (7.1)	1 (7.7)	0
3 viruses^6^	1 (2.0)	3 (6.8)	3 (6.5)	0	0	1 (1.6)	4 (2.4)	3 (9.4)	0	0	1 (7.7)	0
Bacteraemia^7^	3 (6.4)	3 (7.0)	7 (15.2)	2 (25.0)	0	6 (10.2)	11 (7.1)	0	2 (12.5)	1 (7.7)	2 (18.2)	1 (25.0)

1 : Numbers in parenthesis are percentage of total nasopharyngeal aspirates.

2 : Only one virus detected. Numbers in parenthesis are percentage of positive samples for that virus.

3 : Numbers in parenthesis are percentage of samples positive for the specific virus in the column co-infected with the virus(es) in the rows.

4 : Previously-tested by immunofluorescence assay.

5 : Previously-tested previously by nested-PCR.

6 : Including viruses previously-tested by immunofluorescence assay (Influenza A, RSV, PIV, adenovirus) and nested-PCR (hMPV).

7 : 487 patients had specimens available for culture. Bacteria isolated included: hBoV with *Escherichia coli* (n = 2) and *Salmonella* sp (n = 1); WUPyV with *Streptococcus pneumoniae* (n = 2) and *Escherichia coli* (n = 1); KIPyV with *Streptococcus pneumoniae* (n = 3), *Escherichia coli* (n = 3) and *Haemophilus parainfluenzae* (n = 1); CoV-NL63 with *Streptococcus pneumoniae* (n = 2); CoV-OC43 with *Escherichia coli* (n = 3), *Salmonella* sp (n = 1), *Streptococcus viridans* (n = 1) and other *Streptococcus* (n = 1); hRV with *Streptococcus pneumoniae* (n = 6), *Escherichia coli* (n = 3), *Streptococcus viridans* (n = 1) and *Citrobacter freundii* (n = 1); hMPV with *Salmonella* sp (n = 2); PIV with *Streptococcus viridans* (n = 1); Influenza A with *Streptococcus pneumoniae* (n = 1) and *Escherichia coli* (n = 1); Adenovirus with *Pseudomonas aeruginosa* (n = 1).

hBoV: human bocavirus.

WUPyV: polyomavirus-WU.

KIPyV: polyomavirus-KI.

CoV-NL63: coronavirus NL63.

CoV-HKU1: coronavirus HKU1.

CoV-OC43: coronavirus OC43.

CoV-229E: coronavirus 229E.

hRV: human rhinovirus.

RSV: respiratory syncytial virus.

PIV: parainfluenza viruses.

hMPV: human metapneumovirus.

In HIV-uninfected children at least one newly-tested virus was detected in 509 (54.0%) LRTI-episodes, with hRV also being the most common (32.0%), followed by hBoV (13.3%), WUPyV (11.9%), KIPyV (4.8%), CoV-OC43 (3.6%), CoV-NL63 (2.6%), CoV-HKU1 (1.6%) and CoV-229E (0.42%); Table 3. Comparing HIV-uninfected PCV9 and placebo recipients, differences in the prevalence of newly-tested viruses were evident for KIPyV (2.1% vs. 7.4%; p<0.001), CoV-HKU1 (0.21% vs. 2.9%; p = 0.001), CoV-OC43 (2.1% vs. 5.0%; p = 0.017) and hRV (36.2% vs. 27.9%; p = 0.007); Table S3. Of the 302 LRTI-episodes in which hRV was identified, 51.3% had at least one other virus detected, including 13.6% with RSV, 12.3% with WUPyV or hBoV and 2.0% (N = 6) with both WUPyV and hBoV; Table 5. The prevalence of bacteraemia in HIV-uninfected children among those with blood culture results was 2.7% (N = 24/881) of which 12 (50.0%) occurred in the presence of infection with one of the newly-tested viruses and 15 (62.5%) in presence of any of the studied viruses.

**Table 5:** Respiratory viruses co-infections in HIV-uninfected children hospitalized with lower respiratory tract infections.

	hBoV	WUPyV	KIPyV	CoV-NL63	CoV-HKU1	CoV-OC43	CoV-229E	hRV	RSV^4^	hMPV^5^	PIV^4^	Influenza A^4^	Adenovirus^4^
Positive samples^1^	125 (13.3)	112 (12.0)	45 (4.8)	24 (2.6)	15 (1.6)	34 (3.6)	4 (0.42)	302 (32.0)	205 (21.8)	90 (9.7)	44 (4.7)	48 (5.1)	25 (2.7)
Single infections^2^	32 (25.6)	31 (27.7)	10 (22.2)	5 (20.8)	6 (40.0)	8 (23.5)	1 (25.0)	147 (48.7)	128 (62.4)	46 (51.1)	16 (36.4)	27 (56.3)	10 (40.0)
Co-infections with^3^:													
hBoV	-	19 (17.0)	5 (11.1)	5 (23.8)	2 (13.3)	5 (14.7)	0	37 (12.3)	20 (9.8)	8 (10.8)	4 (9.1)	8 (16.7)	9 (36.0)
WUPyV	19 (15.2)	-	5 (11.1)	3 (12.5)	3 (20.0)	7 (20.6)	0	37 (12.3)	14 (6.8)	12 (16.2)	4 (9.1)	7 (14.6)	4 (16.0)
KIPyV	5 (4.0)	5 (4.5)	-	2 (8.3)	1 (6.7)	2 (5.9)	2 (50.0)	15 (5.0)	3 (1.5)	8 (10.8)	6 (13.6)	1 (2.1)	3 (12.0)
CoV-NL63	5 (4.0)	3 (2.7)	2 (4.4)	-	1 (6.7)	0	0	9 (3.0)	6 (2.9)	2 (2.7)	0	1 (2.1)	0
CoV-HKU1	2 (1.6)	3 (2.7)	1 (2.2)	1 (4.2)	-	1 (2.9)	0	2 (0.7)	1 (0.5)	2 (2.7)	1 (2.3)	0	0
CoV-OC43	5 (4.0)	7 (6.3)	2 (4.4)	0	1 (6.7)	-	0	10 (3.3)	6 (2.9)	5 (6.8)	3 (6.8)	1 (2.1)	1 (4.0)
CoV-229E	0	0	2 (4.4)	0	0	0	-	0	1 (0.5)	1 (1.4)	0	0	0
hRV	37 (29.6)	37 (33.0)	15 (33.3)	9 (37.5)	2 (13.3)	10 (29.4)	0	-	41 (20.0)	18 (24.3)	13 (29.5)	10 (20.8)	4 (16.0)
RSV^4^	20 (16.0)	14 (12.4)	3 (6.7)	6 (25.0)	1 (6.7)	6 (17.6)	1 (25.0)	41 (13.6)	-	4 (5.4)	0	0	0
hMPV^5^	8 (6.4)	12 (10.6)	8 (17.8)	2 (8.3)	2 (13.3)	5 (14.7)	1 (25.0)	18 (6.0)	4 (2.0)	-	0	1 (2.1)	0
PIV^4^	4 (3.2)	4 (3.5)	6 (13.3)	0	1 (6.7)	3 (8.8)	0	13 (4.3)	0	0	-	0	0
Influenza A^4^	8 (6.4)	7 (6.2)	1 (2.2)	1 (4.2)	0	1 (3.3)	0	10 (3.3)	0	1 (1.4)	0	-	0
Adenovirus^4^	9 (7.2)	4 (3.5)	3 (6.7)	0	0	1 (2.9)	0	4 (1.3)	0	0	0	0	-
1 viruses^6^	66 (52.8)	53 (46.9)	19 (42.2)	9 (37.5)	5 (33.3)	15 (44.1)	2 (50.0)	118 (39.1)	62 (30.2)	32 (35.6)	25 (56.8)	13 (27.1)	9 (36.0)
2 viruses^6^	25 (20.0)	22 (19.6)	14 (31.1)	10 (41.7)	3 (20.0)	7 (20.6)	1 (25.0)	33 (10.9)	11 (5.4)	7 (7.8)	3 (6.8)	8 (16.7)	6 (24.0)
3 viruses^6^	2 (1.6)	6 (5.3)	2 (4.4)	0	1 (6.7)	4 (11.8)	0	4 (1.3)	4 (2.0)	5 (5.6)	0	0	0
Bacteraemia^7^	0	2 (1.9)	0	1 (4.2)	0	1 (3.0)	0	11 (3.9)	3 (1.6)	1 (1.2)	3 (7.0)	1 (2.1)	0

1 : Numbers in parenthesis are percentage of total nasopharyngeal aspirates.

2 : Only one virus detected. Numbers in parenthesis are percentage of positive samples for that virus.

3 : Numbers in parenthesis are percentage of samples positive for the specific virus in the column co-infected with the virus(es) in the rows.

4 : Previously-tested by immunofluorescence assay.

5 : Previously-tested previously by nested-PCR.

6 : Including previously-tested by immunofluorescence assay (Influenza A, RSV, PIV, adenovirus) and nested-PCR (hMPV).

7 : 881 patients had specimens available for culture. Bacteria isolated included: WUPyV with *Streptococcus pneumoniae* (n = 2); CoV-NL63 with *Micrococcus* (n = 1); CoV-OC43 with *Streptococcus viridans* (n = 1); hRV with *Streptococcus pneumoniae* (n = 4), *Haemophilus influenzae* type b (n = 2), *Klebsiella* sp. (n = 2), *Streptococcus viridans* (n = 1), *Enterococcus faecalis* (n = 1) and *Micrococcus* (n = 1); RSV with *Streptococcus pneumoniae* (n = 1), *Haemophilus influenzae* type b (n = 1) and *Micrococcus* (n = 1); hMPV with *Streptococcus viridans* (n = 1); PIV with *Streptococcus pneumoniae* (n = 1), *Staphylococcus aureus* (n = 1) and *Klebsiella* sp. (n = 1); Influenza A with *Streptococcus pneumoniae* (n = 1).

hBoV: human bocavirus.

WUPyV: polyomavirus-WU.

KIPyV: polyomavirus-KI.

CoV-NL63: coronavirus NL63.

CoV-HKU1: coronavirus HKU1.

CoV-OC43: coronavirus OC43.

CoV-229E: coronavirus 229E.

hRV: human rhinovirus.

RSV: respiratory syncytial virus.

PIV: parainfluenza viruses.

hMPV: human metapneumovirus.

By multivariate analysis, adjusting for PCV-vaccination status, period of collection and age, single infections with a newly-tested virus were more frequent in HIV-infected (30.2%) than HIV-uninfected children (25.5%) (adjusted odds ratio [aOR] 1.3; p = 0.033); Table 3. Also, HIV-infected compared to HIV-uninfected children had a higher prevalence of KIPyV (aOR 2.14; p = 0.002) and CoV-OC43 (aOR 3.67; p<0.001) and a lower prevalence of hBoV (aOR 0.69; p = 0.043) and WUPyV (aOR 0.66; p = 0.035); Table 3. Concurrent bacteraemia and infection with at least one of the newly-tested viruses was more frequent in HIV-infected (7.7%) compared to HIV-uninfected children (2.5%, aOR 3.49; p = 0.001). There were no differences in the frequency of bacteraemia comparing children in whom newly-tested viruses were detected and those without viral detection both in HIV-infected (7.7% vs. 8.0%; p = 0.911) and HIV-uninfected children (2.5% vs. 2.9%; p = 0.713).

### Respiratory viruses and clinical manifestations

When compared to LRTI-episodes associated with the identification of only a single virus the detection of multiple viruses in HIV-infected children was significantly associated with a higher frequency of bronchial breathing (aOR 2.11; p = 0.015) and in HIV-uninfected children with a higher prevalence of cyanosis (aOR 2.50; p = 0.008) and wheezing (aOR 1.55; p = 0.006); Table 6. No differences were detected in the severity of LRTI-episodes with single and multiple viral infections using the RISC score previously developed.

**Table 6:** Clinical and laboratory features of HIV-infected and -uninfected children hospitalized for lower respiratory tract infection by detection of viral co-infections.

	HIV-infected				HIV-uninfected			
	single virus detectedN = 197	multiple virus detectedN = 118	OR (95% CI)p-value	aOR^1^ (95% CI)p-value	single virus detectedN = 467	multiple virus detectedN = 271	OR (95% CI)p-value	aOR^1^ (95% CI)p-value
**Clinical characteristics**								
Fever, N (%)	136 (69.7)	92 (78.0)	1.53 (0.90–2.61)0.114	1.49 (0.87–2.56)0.146	341 (73.5)	194 (71.9)	0.92 (0.66–1.29)0.630	0.90 (0.63–1.27)0.544
Vomit, N (%)	85 (43.4)	51 (43.2)	0.99 (0.63–1.58)0.980	0.91 (0.56–1.47)0.699	209 (44.9)	108 (40.0)	0.82 (0.60–1.11)0.201	0.83 (0.61–1.13)0.244
Seizures, N (%)	3 (1.6)	2 (1.7)	1.10 (0.18–6.69)0.916	1.10 (0.17–7.01)0.916	19 (4.1)	9 (3.4)	0.81 (0.36–1.81)0.607	0.76 (0.33–1.71)0.502
Cyanosis, N (%)	40 (20.6)	23 (19.5)	0.93 (0.53–1.65)0.810	1.03 (0.66–1.87)0.934	16 (3.4)	21 (7.8)	2.361.21–4.600.012	2.501.27–4.900.008
Mean oxygen saturation on room air <90%, N (%)	78 (40.2)	46 (39.7)	0.98 (0.61–1.56)0.924	1.14 (0.69–1.89)0.604	68 (14.7)	43 (15.9)	1.10 (0.73–1.67)0.643	10 (0.72–1.68)0.654
Clinical pneumonia, N (%)	169 (85.8)	107 (90.7)	1.61 (0.77–3.37)0.205	1.56 (0.72–3.35)0.257	233 (49.9)	120 (44.3)	0.80 (0.59–1.08)0.141	0.77 (0.57–1.04)0.090
Severe pneumonia, N (%)	110 (88.7)	76 (92.7)	1.61 (0.59–4.38)0.349	1.67 (0.61–4.61)0.319	232 (82.6)	128 (78.5)	0.77 (0.48–1.25)0.296	0.80 (0.49–1.31)0.373
Wheezing, N (%)	42 (21.3)	19 (16.1)	0.71 (0.39–1.29)0.258	0.69 (0.37–1.28)0.244	262 (56.2)	178 (65.7)	1.49 (1.09–2.03)0.012	1.55 (1.13–2.12)0.006
Bronchial breathing, N (%)	27 (13.7)	29 (24.6)	2.05 (1.14–3.68)0.016	2.11 (1.16–3.85)0.015	13 (2.8)	11 (4.1)	1.48 (0.65–3.35)0.349	1.42 (0.62–3.24)0.403
Mechanical ventilation, N (%)	1 (0.51)	0	1.00^2^	-	6 (1.2)	6 (2.6)	0.85 (0.25–2.85)0.791	0.79 (0.23–2.67)0.704
CXR-AC, N (%)	66 (38.8)	46 (44.7)	1.27 (0.77–2.09)0.342	1.33 (0.78–2.27)0.287	74 (17.4)	46 (19.4)	1.15 (0.76–1.72)0.514	1.13 (0.75–1.70)0.571
Hospital stay ≤2 days, N (%)	88 (44.7)	50 (42.4)	0.91 (0.57–1.44)0.1691	0.85 (0.52–1.39)0.511	336 (72.0)	207 (76.4)	1.26 (0.89–1.78)0.188	1.30 (0.91–1.85)0.153
Hospital stay ≥5 days, N (%)	82 (41.6)	54 (45.8)	1.18 (0.75–1.87)0.473	1.27 (0.79–2.05)0.330	91 (19.5)	44 (16.2)	0.80 (0.54–1.19)0.272	0.80 (0.54–1.20)0.285
Deaths due to LRTI, N (%)	33 (16.8)	16 (13.6)	0.83 (0.44–1.58)0.582	0.94 (0.48–1.82)0.844	5 (1.1)	2 (0.74)	0.69 (0.13–3.57)0.655	0.74 (0.14–3.90)0.727
**Laboratory investigations**								
CRP ≥40 mg/l, N (%)	49 (32.0)	41 (44.1)	1.67 (0.98–2.85)0.058	1.72 (0.98–2.99)0.057	81 (26.2)	38 (21.2)	0.76 (0.49–1.18)0.217	0.71 (0.45–1.12)0.145
PCT ≥2 ng/ml, N (%)	29 (25.2)	26 (37.1)	1.75 (0.92–3.33)0.087	1.77 (0.92–3.41)0.087	45 (16.4)	24 (15.5)	0.94 (0.55–1.61)0.811	0.82 (0.47–1.44)0.496
Bacteraemia, N (%)	12 (6.6)	10 (8.8)	1.37 (0.57–3.28)0.480	1.39 (0.58–3.35)0.465	8 (1.9)	7 (2.7)	1.47 (0.53–4.10)0.464	1.54 (0.55–4.33)0.413

Viral detection including newly-tested and previously-tested viruses.

1 : Adjusted odds ratio (aOR) adjusted for age, year of sampling and whether received 9-valent pneumococcal conjugate vaccine or placebo.

2 : p-value not adjusted.

OR: odds ratio.

CRP: C-reactive protein.

PCT: Procalcitonin.

CXR-AC: Alveolar consolidation on chest x-ray.

In both HIV-infected and –uninfected children with bacteria isolated from blood, there were no significant differences in the clinical and laboratory characteristics between children in whom viruses were detected and children without any virus detected. The same was observed restricting the analysis to *Streptococcus pneumoniae* isolation (data not shown).

## Discussion

To our knowledge, this study provides the most in-depth analysis of the prevalence of hRV and some of the newly-discovered respiratory viruses in HIV-infected children. Our study identified hRV to be the most frequently detected virus both in HIV-infected and –uninfected children followed by CoV-OC43 in HIV-infected and hBoV in HIV-uninfected. Most cases of hospitalizations associated with single infections overall were noted for hRV and RSV, the rate of co-infections was high in children infected with the other newly-discovered viruses.

Very few viral aetiology studies have been conducted in Africa: in a Mozambican study of virus-associated acute respiratory infections (ARI) in infants with an estimated 3–5% HIV prevalence, the most frequently detected viruses were hRV (26%), influenza (15%) and adenovirus (14%) [27]. A recent study from South Africa on children with ARI requiring medical attention, 61% (N = 383) being HIV-infected or HIV-seropositive, also reported that hRV was the most frequently detected virus (33%) followed by RSV (30%), PIV (8%) and hBoV (6%) [21]. In the South African study hospitalized children with respiratory disease had higher hRV detection rates compared to healthy children (36% vs. 19%; p = 0.047) which may indicate a causality effect of hRV in disease [21]. In Kenya in older children (5–17 years old), despite hRV being the most commonly detected virus (38%) in patients presenting with ARI, its detection rate was similar among asymptomatic controls [28]. Indeed two other studies in Kenya found that with the exception of RSV, viral detection in the nasopharynx did not have a significant association with pneumonia in hospitalized children under 5 years [29] or under 12 years, [30] compared to children both without symptoms of URTI or with respiratory symptoms but not meeting any criteria for pneumonia.

It is uncertain as to what the impact of HIV infection on the duration of shedding of these viruses might be. An analysis of serial respiratory samples from otherwise healthy children detected shedding of CoV-NL63 for up 21 days [31], hRV up to 41 days and of hBoV up to 44 days [32]. Detection of CoV-HKU1 and CoV-229E in respiratory specimens of transplanted children were also reported for at least 38 days and 11 weeks, respectively, possibly suggesting that immunocompromised children may have a prolonged duration of shedding of these viruses [33], [34]. Such prolonged shedding in immunocompromised individuals such as HIV-infected children, may result in a greater frequency of co-incidental identification of these viruses when investigated for respiratory illness, affect the seasonal occurrence of the viruses as well as potentially present a threat to greater nosocomial transmission of these viruses because of higher incidence of hospitalization of HIV-infected children.

The casual relationship between viral DNA detection, development of disease and mixed infections is very complex. In the case of hBoV serological studies have shown that the presence of viral DNA in respiratory samples was not proof of an acute primary infection and that prolonged viral shedding could be an explanation for viral detection at high rates in asymptomatic controls [35]. Enrolment of healthy non-hospitalized children and quantification of viral loads may help to elucidate the problem of co-infections and is currently being under-taken in a multi-centre study on aetiology of pneumonia in children [36]. Contradictory findings on whether infection with multiple viruses contribute to disease severity in hospitalized children have been reported, with some studies reporting an increased severity among children with viral co-infections [21], [37] and others finding decrease severity associated with co-infections [38]. In some studies no differences were observed [39]. We found that multiple viral infections were associated with bronchial breathing in HIV-infected children and with cyanosis and wheezing in HIV-uninfected children. Bacterial co-infections can also increase the severity of viral diseases [40]. Furthermore, in children with invasive pneumococcal disease, superimposed viral co-infections are common and may lead to higher mortality rates [41]. Of the participants in our study from whom bacterial cultures performed, no differences in clinical and laboratory characteristics were observed between children with or without viral infections. Our analysis, however, included PCV9 vaccinees who probably present with less severe LRTI cases since it was shown before that PCV9 vaccination was associated with a decrease in LRTI viral-associated hospitalizations [5], [23]. We detected a high prevalence of bacterial co-infections with the newly-tested viruses especially in HIV-infected children (8%), what may have contributed to the higher C-reactive protein and procalcitonin levels, the longer hospital stay and the higher mortality in HIV-infected children compared to HIV-uninfected.

Our high co-infection detection rates are similar to other molecular detection studies of paediatric respiratory samples, with coronaviruses (40–75%) [42], [43], hBoV (up to 78%) [13], [44], [45] and polyomaviruses (68–79%) [46]–[48]. Our results may, however, have under-estimated the true prevalence of viral co-infections since the previously-studied viruses were tested by immunofluorescence [5] or conventional PCR [23]. Although the sensitivity of the conventional methods is reported to be high, comparative studies have shown that RT-PCR is considerably more sensitive for detection of respiratory viruses [21], [49]. Moreover the specimens tested by immunofluorescence were initially screened for RSV and only if negative were further tested for influenza A/B, PIV I–III and adenovirus [5].

Limitations of our study include that this was a post-hoc analysis and only 70% of specimens collected during the study period were available for further testing [24]. Compared with the specimens available for RT-PCR the specimens unavailable were collected from younger children and were more likely to have tested positive for a previously-studied virus. This selection bias may have also contributed to an under-estimation of the number of co-infections. Along the same line RSV-associated LRTI are more common among younger children [21], [39], for whom we had less available samples increasing our uncertainty on the prevalence of RSV co-infections with the newly-tested viruses.

An equal proportion of PCV9- and placebo-recipients hospitalized with LRTI were investigated in this study and our analyses were adjusted for the initial intervention arm. Further detailed analyses on the effect of PCV9 on the incidence of hospitalization associated with the individual newly-tested viruses will be reported on in future.

Although the development of new diagnostic techniques allows more detailed investigation and has led to the discovery of a number of new putative respiratory pathogens, the role for some of these potential pathogens in respiratory illness remains speculative in the absence of fulfilling Koch's postulates for causality. In the present study we demonstrate that at least one respiratory virus was identified in the majority of HIV-infected and HIV-uninfected children hospitalized for LRTI. The difficulty in attributing disease causality to specific viruses due to the high frequency of viral co-infection suggests a possible synergy among different pathogens during childhood LRTI.

## Supporting Information

Table S1 Primer and probe sequences for real-time reverse transcriptase–polymerase chain reaction viral detection used in the study.
(DOCX)

Table S2 Demographic, clinical and laboratory features which differed significantly between lower respiratory tract infection episodes in which samples were available or unavailable for further testing in the current study.
(DOCX)

Table S3 Viral prevalence according to initial study arm and HIV status.
(DOCX)
