# Supplementary material for: Clinical Epidemiology of Bocavirus, Rhinovirus, Two Polyomaviruses and Four Coronaviruses in HIV-Infected and HIV-Uninfected South African Children
Source: PLoS One. 2014 Feb 3;9(2):e86448. doi: 10.1371/journal.pone.0086448 (PMC3911925; doi:10.1371/journal.pone.0086448)
Supplement: Table S1 — Primer and probe sequences for real-time reverse transcriptase–polymerase chain reaction viral detection used in the study. (DOCX) [file pone.0086448.s001.docx]

**Table S1. Primer and probe sequences for real-time reverse transcriptase–polymerase chain reaction viral detection used in the study**

|  | Sequences  (5’ → 3’) | Target gene |
| --- | --- | --- |
| Human Rhinovirus | F1: GCC TGC GTG GCT GCC  R1: CCT GCG TGG CGG CC  F2: ACG GAC ACC CAA AGT AGT TGG T  R2: ACG GAC ACC CAA AGT AGT CGG T  Probe1: FAM-TCC GGC CCC TGA ATG TGG CTA A-MGB  Probe2: FAM-TCC GGC CCC TGA ATG CGG CTA A-MGB | 5’UTR |
| Human Bocavirus | F: GGA AGA GAC ACT GGC AGA CAA  R: GGG TGT TCC TGA TGA TAT GAG C  Probe: FAM- CTG CGG CTC CTG CTC CTG TGA T -TAMRA | Nonstructural protein 1 |
| Polyomavirus-WU | F: GGC ACG GCG CCA ACT  R: CCT GTT GTA GGC CTT ACT TAC CTG TA  Probe: FAM-TGC CAT ACC AAC ACA GCT GCT GAG C-TAMRA | DNA Binding Protein Gp5 |
| Polyomavirus-KI | F: ACC TGA TAC CGG CGG AAC T  R: CGC AGG AAG CTG GCT CAC  Probe: VIC-CCA CAC AAT AGC TTT CAC TCT TGG CGT GA-TAMRA | Non-translated region |
| Human coronavirus-NL63 | F: GCG TGT TCC TAC CAG AGA GGA  R: GCT GTG GAA AAC CTT TGG CA  Probe: VIC-ATG TTA TTC AGT GCT TTG GTC CTC GTG AT-TAMRA | Nucleoprotein (N) |
| Human coronavirus-HKU1 | F: CGC CTG GTA CGA TTT TGC C  R: GAA CGA TTA TTG GGT CCA CGT G  Probe: VIC- TGTTGA AGG CTC AGG AAG GTC TGC TTC TAA-TAMRA | Nucleoprotein (N) |
| Human coronavirus-OC43 | F: CGA TGA GGC TAT TCC GAC TAG GT  R: CCT TCC TGA GCC TTC AAT ATA GTA ACC  Probe: FAM-TCC GCC TGG CAC GGT ACT CCC T-TAMRA | Nucleoprotein (N) |
| Human coronavirus-229E | F: CAG TCA AAT GGG CTG ATG CA  R: AAA GGG CTA TAA AGA GAA TAA GGT ATT CT  Probe: VIC-CCC TGA CGA CCA CGT TGT GGT TCA-TAMRA | Nucleoprotein (N) |
