# Supplementary material for: Clinical Epidemiology of Bocavirus, Rhinovirus, Two Polyomaviruses and Four Coronaviruses in HIV-Infected and HIV-Uninfected South African Children
Source: PLoS One. 2014 Feb 3;9(2):e86448. doi: 10.1371/journal.pone.0086448 (PMC3911925; doi:10.1371/journal.pone.0086448)
Supplement: Table S2 — Demographic, clinical and laboratory features which differed significantly between lower respiratory tract infection episodes in which samples were available or unavailable for further testing in the current study. (DOCX) [file pone.0086448.s002.docx]

**Table S2. Demographic, clinical and laboratory features which differed significantly between lower respiratory tract infection episodes in which samples were available or unavailable for further testing in the current study**

|  |  | **NPA done**  N=2094  HIV-infected=687  HIV-uninfected=1407 | **Available for further PCR testing**  N=1460  HIV-infected=517  HIV-uninfected=943 | **Unavailable for further PCR testing**  N=634  HIV-infected=170  HIV-uninfected=464 | **p-value^1^** |
| --- | --- | --- | --- | --- | --- |
|  | Overall | 9 (1-24) | 10 (1-24) | 8 (1-24) | <0.001 |
| Median age in | HIV-infected | 8 (1-24) | 9 (1-24) | 8 (1-23) | 0.454 |
| months, (range) | HIV-uninfected | 10 (1-24) | 11 (1-23) | 8 (1-24) | <0.001 |
|  | Overall | 868 (41.6) | 624 (42.9) | 244 (38.6) | 0.068 |
| Vomit, | HIV-infected | 277 (40.6) | 209 (40.7) | 68 (40.2) | 0.922 |
| N (%) | HIV-uninfected | 591 (42.1) | 415 (44.1) | 176 (38.0) | 0.030 |
|  | Overall | 216 (10.4) | 165 (11.4) | 51 (8.1) | 0.025 |
| Cyanosis, | HIV-infected | 152 (22.3) | 122 (23.8) | 30 (17.8) | 0.102 |
| N (%) | HIV-uninfected | 64 (4.6) | 43 (4.6) | 21 (4.6) | 0.984 |
|  | Overall | 468 (25.2) | 342 (26.6) | 126 (22.1) | 0.041 |
| CXR-AC, | HIV-infected | 243 (41.5) | 189 (43.2) | 54 (36.5) | 0.155 |
| N (%) | HIV-uninfected | 225 (17.7) | 153 (18.1) | 72 (17.1) | 0.673 |
|  | Overall | 14.1 (1-482) | 15 (1-464) | 12 (1-482) | 0.003 |
| Median CRP | HIV-infected | 18 (1-464) | 18 (1-464) | 17 (1-324) | 0.329 |
| mg/l, (range) | HIV-uninfected | 13 (1-482) | 14 (1-446.2) | 11 (1-482) | 0.007 |
|  | Overall | 0.23 (0.01-302) | 0.26 (0.01-302) | 0.15 (0.01-191.4) | 0.006 |
| Median PCT | HIV-infected | 0.44 (0.01-302) | 0.47 (0.01-302) | 0.28 (0.08-161.1) | 0.121 |
| ng/ml, (range) | HIV-uninfected | 0.14 (0.01-191.4) | 0.17 (0.01-150.1) | 0.12 (0.01-191.4) | 0.228 |
| Other virus | Overall | 755 (36.1) | 487 (33.4) | 268 (42.3) | <0.001 |
| previously | HIV-infected | 125 (18.2) | 80 (15.5) | 45 (26.5) | 0.001 |
| detected, N (%) | HIV-uninfected | 630 (44.8) | 407 (43.2) | 223 (48.1) | 0.085 |

^1^: Chi-square or Fischer test and Mann-Whitney test comparing nasopharyngeal aspirate (NPA) available for RT-PCR testing and NPA unavailable for RT-PCR.

Vomit: 2087 participants with available information; available for further RT-PCR testing 1455; unavailable for further RT-PCR testing 632.

Cyanosis: 2085 participants with available information; available for further RT-PCR testing 1454; unavailable for further RT-PCR testing 631.

Alveolar consolidation on chest x-ray (CXR-AC): 1854 participants with available information; available for further RT-PCR testing 1285; unavailable for further RT-PCR testing 569.

C-reactive protein (CRP): 1472 participants with available information; available for further RT-PCR testing 1035; unavailable for further RT-PCR testing 437.

Procalcitonin (PCT): 1230 participants with available information; available for further RT-PCR testing 865; unavailable for further RT-PCR testing 365.

Tested for other viruses: 2092 specimens; available for further RT-PCR testing 1058; unavailable for further RT-PCR testing 634.
