# Supplementary material for: Clinical Epidemiology of Bocavirus, Rhinovirus, Two Polyomaviruses and Four Coronaviruses in HIV-Infected and HIV-Uninfected South African Children
Source: PLoS One. 2014 Feb 3;9(2):e86448. doi: 10.1371/journal.pone.0086448 (PMC3911925; doi:10.1371/journal.pone.0086448)
Supplement: Table S3 — Viral prevalence according to initial study arm and HIV status. (DOCX) [file pone.0086448.s003.docx]

**Table S3. Viral prevalence according to initial study arm and HIV status**

|  |  | **Overall, n (%)**  **N=1460**  **PCV9=699**  **Placebo=761** | **HIV-infected, n (%)**  **N=517**  **PCV9=232 Placebo=285** | **HIV-uninfected, n (%)**  **N=943**  **PCV9=467 Placebo=476** | **p-value^1^** |
| --- | --- | --- | --- | --- | --- |
|  | Overall | 174 (11.9) | 49 (9.5) | 125 (13.3) | 0.033 |
| hBoV | PCV9 | 87 (12.5) | 29 (12.5) | 58 (12.4) | 0.976 |
|  | Placebo | 87 (11.4) | 20 (7.0) | 67 (14.1) | 0.003 |
|  | p-value^2^ | 0.550 | 0.034 | 0.453 |  |
|  | Overall | 156 (10.7) | 44 (8.5) | 112 (11.9) | 0.046 |
| WUPyV | PCV9 | 72 (10.3) | 26 (11.2) | 46 (9.9) | 0.578 |
|  | Placebo | 84 (11.0) | 18 (6.3) | 66 (13.9) | 0.001 |
|  | p-value^2^ | 0.649 | 0.047 | 0.057 |  |
|  | Overall | 91 (6.2) | 46 (8.9) | 45 (4.8) | 0.002 |
| KIPyV | PCV9 | 27 (3.9) | 17 (7.3) | 10 (2.1) | 0.001 |
|  | Placebo | 64 (8.4) | 29 (10.2) | 35 (7.4) | 0.175 |
|  | p-value^2^ | <0.001 | 0.258 | <0.001 |  |
|  | Overall | 33 (2.3) | 9 (1.7) | 24 (2.6) | 0.323 |
| CoV-NL63 | PCV9 | 19 (2.7) | 4 (1.7) | 15 (3.2) | 0.328 |
|  | Placebo | 14 (1.8) | 5 (1.8) | 9 (1.9) | 1.0 |
|  | p-value^2^ | 0.259 | 1.0 | 0.198 |  |
|  | Overall | 22 (1.5) | 7 (1.4) | 15 (1.6) | 0.723 |
| CoV-HKU1 | PCV9 | 4 (0.57) | 3 (1.3) | 1 (0.21) | 0.109 |
|  | Placebo | 18 (2.4) | 4 (1.4) | 14 (2.9) | 0.222 |
|  | p-value^2^ | 0.005 | 1.0 | 0.001 |  |
|  | Overall | 97 (6.6) | 63 (12.2) | 34 (3.6) | <0.001 |
| CoV-OC43 | PCV9 | 38 (5.4) | 28 (12.1) | 10 (2.1) | <0.001 |
|  | Placebo | 59 (7.8) | 35 (12.3) | 24 (5.0) | <0.001 |
|  | p-value^2^ | 0.076 | 0.942 | 0.017 |  |
|  | Overall | 4 (0.27) | 0 | 4 (0.42) | 0.304 |
| CoV-229E | PCV9 | 1 (0.14) | 0 | 1 (0.21) | 1.0 |
|  | Placebo | 3 (0.39) | 0 | 3 (0.63) | 0.296 |
|  | p-value^2^ | 0.359 | - | 0.624 |  |
|  | Overall | 466 (31.9) | 164 (31.7) | 302 (32.0) | 0.905 |
| hRV | PCV9 | 241 (34.5) | 72 (31.0) | 169 (36.2) | 0.177 |
|  | Placebo | 225 (29.6) | 92 (32.3) | 133 (27.9) | 0.204 |
|  | p-value^2^ | 0.044 | 0.762 | 0.007 |  |
|  | Overall | 783 (53.6) | 274 (53.0) | 509 (54.0) | 0.720 |
| At least 1 new virus | PCV9 | 370 (52.9) | 122 (52.6) | 248 (53.1) | 0.897 |
| detected | Placebo | 413 (54.3) | 152 (53.3) | 261 (54.8) | 0.688 |
|  | p-value^2^ | 0.609 | 0.866 | 0.595 |  |
|  | Overall | 1053 (72.1) | 315 (60.9) | 738 (78.3) | <0.001 |
| At least 1 virus | PCV9 | 500 (71.5) | 141 (60.8) | 359 (76.9) | <0.001 |
| detected^3^ | Placebo | 553 (72.7) | 174 (61.1) | 379 (79.6) | <0.001 |
|  | p-value^2^ | 0.628 | 0.949 | 0.306 |  |
|  | Overall | 389 (26.6) | 118 (22.8) | 271 (28.7) | 0.015 |
| Multiple virus^3^ | vaccine | 183 (26.2) | 57 (24.6) | 126 (27.0) | 0.495 |
|  | PCV9 | 206 (27.1) | 61 (21.4) | 145 (30.5) | 0.006 |
|  | Placebo | 0.701 | 0.394 | 0.268 |  |

^1^: Chi-square or Fischer test comparing HIV groups.

^2^: Chi-square or Fischer test comparing 9-valent pneumococcal conjugated vaccine (PCV9) and placebo arms.

^3^: Including virus previously-tested by immunofluorescence assay (RSV, Influenza A, PIV I-III and adenovirus) and nested-PCR (hMPV).
